# Rad51, friend or foe?

**DOI:** 10.7554/eLife.00914

**Published:** 2013-06-11

**Authors:** Sue Mei Tan-Wong, Nick J Proudfoot

**Affiliations:** 1 **Sue Mei Tan-Wong** is at the Sir William Dunn School of Pathology, University of Oxford, Oxford, United Kingdomsue.wong@path.ox.ac.uk; 2 **Nick J Proudfoot** is an *eLife* reviewing editor, and is at the Sir William Dunn School of Pathology, University of Oxford, Oxford, United Kingdomnicholas.proudfoot@path.ox.ac.uk

**Keywords:** R loops, RNA-DNA hybrids, Rad51, Genome Instability, DNA repair, S. cerevisiae

## Abstract

A protein long recognized for its role in DNA repair has now paradoxically been implicated in DNA damage.

**Related research article** Wahba L, Gore SK, Koshland D. 2013. The homologous recombination machinery modulates the formation of RNA-DNA hybrids and associated chromosome instability. *eLife* **2**:e00505. doi: 10.7554/eLife.00505

**Image** Cartoon showing one way in which Rad51 (blue) could promote the formation of RNA-DNA hybrids

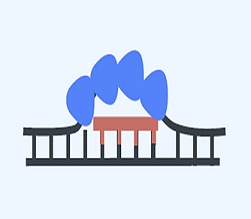

Imagine your frustration if, in the event of a breakdown, the emergency mechanic not only failed to repair your vehicle but actually damaged it even further. A similar situation can arise with our own DNA which, when damaged, is subjected to a system of rigorous checks and balances by the DNA repair machinery. However, DNA can also be damaged through the actions of structures called R-loops. These are RNA-DNA hybrids formed when an RNA transcript invades the DNA double helix, and they are a natural byproduct of the transcription process (Aguilera and Garcia-Muse, 2012). Now, in *eLife*, Douglas Koshland at the University of California, Berkeley, and co-workers reveal that proteins normally involved in DNA repair can also facilitate the formation of R-loops, thus increasing DNA damage (Wahba et al., 2013).

Initial work in bacteria showed that the repair protein RecA (Kasahara et al., 2000) can potentiate the formation of R-loops in the absence of active transcription. Building on this study, Koshland and colleagues—including Lamia Wahba, who is also at Johns Hopkins University, and Steven Gore—report three sets of experiments on the yeast counterpart of RecA, which is known as Rad51. Firstly, they provide evidence that Rad51 is also involved in R-loop formation. Secondly, they show that Rad51 is already present at R-loops before DNA damage occurs, suggesting that it may be oncogenic (i.e., have the potential to cause cancer). Thirdly, through elegant experimentation, they challenge the dogma that R-loop formation must invariably occur at the site of transcription (*cis*-acting) by revealing that R-loops can also be formed by RNA invading the DNA duplex at a remote genomic location (*trans*-acting).

**Figure 1.**
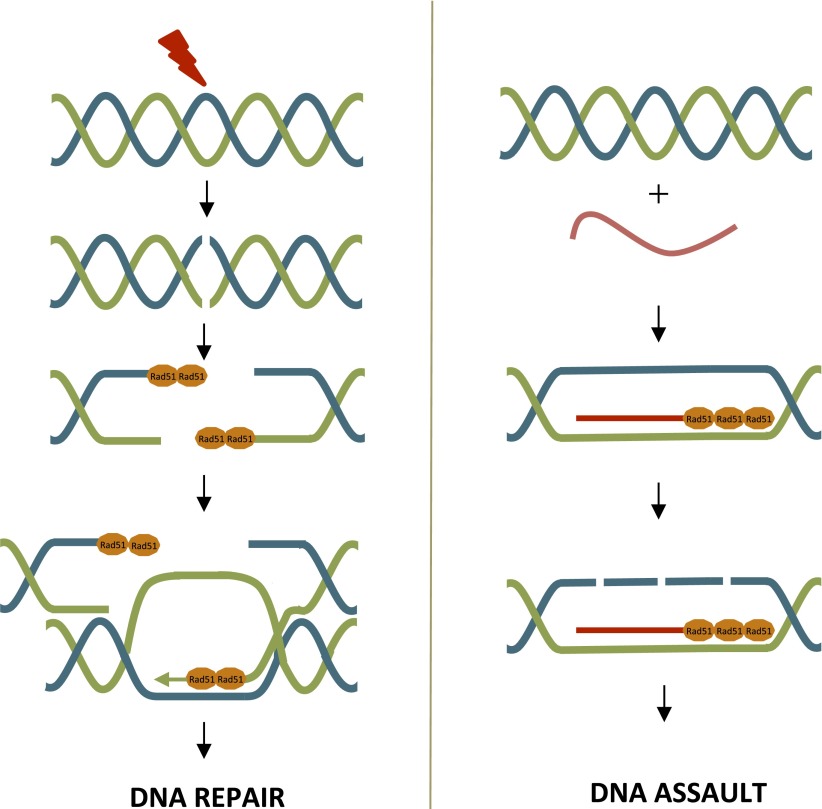
Rad51 can contribute to DNA repair or DNA assault. **Left**: DNA repair through homologous recombination, where one strand is used as a template to repair the other. DNA damage (red bolt) leads to double-strand breaks. At the break point, one strand of the DNA is slightly longer than the other and Rad52 (not shown) promotes the loading of Rad51 (orange) onto these single-strand overhangs. This initiates the process of DNA repair via homologous recombination. Rad51 is removed from the single-strand overhangs by the negative regulator Srs2 (not shown) before the final stages of DNA repair. **Right**: Rad51 can promote the formation of DNA-RNA hybrids (R-loops), which can damage DNA. R-loops form when Rad51 binds repeatedly to RNA (red lines). Srs2 (not shown) inhibits R-loop formation by removing Rad51.

Wahba et al. show that the accumulation of R-loops is dependent on Rad51 activity: in the absence of Rad51, aberrant transcription does not result in the formation of R-loops and the genome remains intact. To measure the destabilizing effects of R-loops on DNA, Wahba et al. used a yeast artificial chromosome (or YAC), which they introduced into the yeast nucleus. They found that Rad51 does not work alone but is regulated by other factors: removing a positive protein regulator of Rad51, called Rad52, blocks the formation of R-loops, whereas removing a negative protein regulator, Srs2, increases their formation.

Koshland and colleagues reveal, in addition, that Rad51 localizes to R-loops. Previous work has shown that Rad51 binds at break points in DNA to initiate DNA repair (Sugawara et al., 2003). Moreover, mammalian Rad51 is known to help cells resist the damaging effects of ionizing radiation, chemotherapeutic agents, and the spontaneous breaks and aberrations that occur during DNA replication. A natural assumption therefore is that the high levels of Rad51 expression seen in tumour cells (Klein, 2008) reflect the protein’s role in DNA repair; in other words, that Rad51 may act as a tumour suppressor.

So what is Rad51 doing at R-loop sites? To address this question, Wahba et al. studied the deposition of a histone called H2AX—a well-defined marker for the formation of double-strand DNA breaks—and compared the timing of this event with the accumulation of Rad51 at an R-loop site. They confirmed that Rad51 was present prior to DNA damage, suggesting that rather than contributing to repair, Rad51 could be driving R-loop-mediated genome instability in cancer cells, and promoting tumour biogenesis.

Having established that Rad51 induces the formation of R-loops, and drawing encouragement from bacterial work, Koshland and colleagues went on to show that R-loop formation is not directly dependent on the act of transcription. To do this, they created yeast cells that contained two copies of a particular DNA sequence—one in a YAC and the other in one of the yeast’s own chromosomes—and placed the latter under the control of a transcriptional switch. When the switch was triggered, the induced chromosomal transcript—with the help of Rad51—invaded the homologous DNA in the YAC. This resulted in the formation of an R-loop, distinct and distant from the original site of transcription. And sure enough, this *trans* induced R-loop destabilized the YAC DNA.

It is noteworthy that R-loops are not always deleterious. As obligatory intermediates, they are essential for mitochondrial DNA replication (Pohjoismaki et al., 2010) and immunoglobulin gene class-switch recombination (Yu et al., 2003)—a complex process in which antibody genes genetically rearrange during the development of immune cells called B cells. They are also implicated in transcription termination (Skourti-Stathaki et al., 2011) and in transcription activation of CpG island promoters (Ginno et al., 2012). However, when R-loops occur out of their natural context and pose a threat to cell survival, all is not lost. Cells have the innate capacity to counteract such hazardous structures. One line of defense is Srs2, which can remove Rad51 from DNA. Other options include Sen1 helicase, an enzyme that can unwind R-loops formed in the wake of transcription (Mischo et al., 2011), and RNAse H, which can degrade RNA that is base-paired with DNA in R-loops (Cerritelli and Crouch, 2009). All of these proteins serve to protect the cellular genome from destruction.

Exploring and harnessing the potential applications of Rad51-dependent R-loop formation is an exciting prospect. The current findings have intriguing parallels with the CRISPR system (Horvath and Barrangou, 2013), which is a mechanism used by bacteria and archaea to defend themselves against invading phages. In brief, short DNA fragments from phage genomes are integrated into the bacterial or archael genome within the CRISPR locus, and transcripts from the CRISPR regions then form R-loops with the infecting phage DNA. These R-loops recruit enzymes to destroy the phage genome. Engineered CRISPR-derived constructs have been used to successfully target specific DNA sequences for replacement or modification in mammalian cells (Wang et al., 2013). Koshland and colleagues now potentially add Rad51 to the arsenal available for genetic search and destroy missions. Consequently, their work may have broad implications for synthetic biology, gene function studies and gene therapy.
